# Family-focused intervention programme to foster adolescent mental health and well-being: protocol for a multicountry cluster randomised factorial trial (FLOURISH Phase 2)

**DOI:** 10.1136/bmjopen-2024-094085

**Published:** 2025-02-07

**Authors:** Antonio Piolanti, Janina Mueller, Franziska Waller, Nina Heinrichs, Judit Simon, Yulia Shenderovich, Swetha Sampathkumar, Dennis Wienand, Marija Raleva, Ivo Kunovski, Viorel Babii, Xiang Zhao, Graham Moore, Heather M Foran

**Affiliations:** 1 Department of Health Psychology, University of Klagenfurt, Klagenfurt, Austria; 2 Department of Psychology, Bielefeld University, Bielefeld, Germany; 3 Department of Health Economics, Center for Public Health, Medical University of Vienna, Vienna, Austria; 4 Department of Psychiatry, University of Oxford, Warneford Hospital, Oxford, UK; 5 Centre for Development, Evaluation, Complexity, and Implementation in Public Health Improvement (DECIPHer), School of Social Sciences, Cardiff University, Cardiff, UK; 6 Wolfson Centre for Young People's Mental Health, Cardiff University, Cardiff, UK; 7 Institute for Marriage, Family, and Systemic Practice - ALTERNATIVA, Skopje, North Macedonia; 8 University Clinic of Psychiatry, St Cyril and Methodius University, Skopje, North Macedonia; 9 Asociatia Obsteasca Sanatate Pentru Tineri (Health for Youth Association), Chisinau, Republic of Moldova

**Keywords:** Adolescents, MENTAL HEALTH, Implementation Science

## Abstract

**Introduction:**

Adolescent mental health problems represent a significant global health issue, particularly in low- and middle-income countries, such as the Republic of North Macedonia and the Republic of Moldova. Effective and scalable interventions are urgently needed to address these challenges.

**Methods and analysis:**

This protocol outlines a multicountry cluster randomised factorial trial, implemented according to the multiphase optimisation strategy (Phase 2), which evaluates the effectiveness and costs of three add-on components for the Parenting for Lifelong Health for Parents and Teens programme: adolescent mental health tools based on UNICEFs Helping Adolescents Thrive comics, adolescent peer support based on UNICEFs ‘I Support My Friends’ intervention and engagement booster designed to enhance attendance and programme completion through incentives. The study will recruit 720 families and involve 64 clusters in North Macedonia and Moldova. Primary outcomes will include adolescent internalising problems and social support, family functioning and attendance during the programme. Secondary outcomes will assess broader aspects of mental health among caregivers and adolescents, as well as implementation and cost outcomes. Data will be collected at baseline and postintervention, approximately, 8 weeks later. Statistical analyses will include regression models to assess the main and interaction effects of the intervention components and cost analyses.

**Ethics and dissemination:**

The study received ethical approval from the University of Klagenfurt in Austria (approval number: 2023–013), the Medical Faculty at St. Cyril and Methodius University in North Macedonia (approval number: 03-2144/4) and the National Committee of Ethical Expertise for Clinical Trials in Moldova (approval number: 1476). The results will be disseminated through peer-reviewed journals, conferences, webinars in multiple languages, regional forums, stakeholder meetings with policymakers and practitioners, public communication through media engagement and open access platforms, including data sharing and early release of findings.

**Trial registration details:**

Trial registration: NCT06562244; Project page: https://www.flourish-study.org/about.html

**Strengths and limitations of this study**

- The study employs a multicountry cluster randomised factorial trial, according to the optimisation phase of the multiphase optimisation strategy framework, which allows for a thorough evaluation of add-on intervention components and their interaction effects on primary outcomes, such as adolescent internalising symptoms, social support, family functioning and programme attendance, as well as secondary outcomes related to broader mental health impacts on caregivers and adolescents.
- The intervention has been carefully adapted to the local contexts of North Macedonia and Moldova, ensuring cultural relevance and increasing the likelihood of successful implementation and participant engagement.
- The intervention is designed to prioritise scalability (eg, brief, easy to disseminate, no licensing cost for materials and small engagement boosters), which facilitates broader implementation and sustainability in low- and middle-income countries.
- While participants and assessors are initially blinded to group assignments and assessors are blinded at postassessment, complete blinding of participants and facilitators is not maintained throughout the study due to the psychosocial nature of the intervention.

## Introduction

 Adolescent mental health is a critical global concern, with estimates indicating that approximately one in seven teenagers worldwide presents psychological problems.^1^ Furthermore, research indicates that one-thirds of individuals experience their first mental disorder before the age of 14, increasing the risk of future mental disorders in adulthood.^2 3^ Poor mental health during adolescence is associated with long-term negative outcomes, such as lower educational attainment, poor physical health and reduced quality of life.^4 5^ The economic impact of untreated adolescent mental health issues is significant, leading to higher costs for healthcare and social services.^6^

In low- and middle-income countries (LMICs), such as the Republic of North Macedonia and the Republic of Moldova (henceforth, North Macedonia and Moldova), adolescents face additional developmental challenges due to heightened socioeconomic stressors, adverse childhood experiences and limited access to mental health services.^7 8^ Moreover, containment measures of the COVID-19 pandemic have placed additional stress on adolescents and their families, leading to increased abuse and neglect.^9^ North Macedonia and Moldova have also experienced an influx of refugees due to the war in Ukraine, many of whom are parents with children facing unique challenges, such as processing of war-related experiences, displacement and challenges related to integrating into new communities.^10 11^

Target 3 of the 2030 UN Agenda for Sustainable Development Goals aims to ensure healthy lives and well-being for all age groups, emphasising the necessity of programmes that promote adolescent mental health, especially in LMICs.^12^ Parenting interventions are recommended as critical strategies to strengthen adolescent–caregiver relationships, enhance parenting styles and improve interpersonal and emotion regulation skills.^13^ One such suite of programmes is the ‘Parenting for Lifelong Health’ (PLH), developed in collaboration with UNICEF, WHO and other international agencies and researchers. These are group-based behavioural programmes that focus on enhancing psychosocial skills and family relationships, targeting different age groups.^14^ The PLH for Young Children, targeting caregivers of children aged 2–9, has been successfully implemented across various cultural settings (Foran *et al* and Heinrichs *et al*).^15^,^17^ Furthermore, the PLH for Parents and Teens programme has been tested in South Africa, supporting its potential for broader applications among adolescents.^18 19^

The systematic optimisation of family programmes is crucial to enhance scalability and sustainability in low-resource settings.^20^ The multiphase optimisation strategy (MOST) provides a critical framework for optimising and evaluating complex behavioural interventions and consists of three phases.^21 22^ The preparation phase (Phase 1) involves developing a conceptual model for the intervention and pilot testing. The optimisation phase (Phase 2) uses methods, such as factorial designs, to test different combinations of intervention components to identify the most effective and efficient configuration. Finally, the evaluation phase (Phase 3) involves conducting a randomised controlled trial (RCT) to assess the optimised intervention’s effectiveness. Using the MOST framework, previous research in Eastern Europe found PLH for Young Children to improve parenting behaviours and child mental health (Foran *et al* and Heinrichs *et al*).^16^ Building on this research, the ‘family-focused adolescent and lifelong health promotion’ (FLOURISH) project aims to adapt, optimise and evaluate an intervention package based on the PLH for Parents and Teens programme targeting adolescents aged 10–14 years and their caregivers in North Macedonia and Moldova. This intervention package seeks to enhance adolescent mental health and well-being by developing skills in both teens and caregivers. Phase 1 of the FLOURISH project aimed to assess the feasibility and preliminary efficacy of the adapted PLH programme.^23^ Alongside local contextual adaptations to the PLH for Parents and Teens programme, the intervention incorporated three additional components to improve programme participation, retention rates and effectiveness. These components included adolescent mental health tools based on UNICEFs Helping Adolescents Thrive (HAT) comics, adolescent peer support based on UNICEFs ‘I Support My Friends’ intervention and engagement boosters. The inclusion of these components is critical for several reasons. First, the HAT framework provides structured tools that can help adolescents manage stress and develop essential emotion regulation skills.^24^ Second, peer support can enhance adolescents’ sense of belonging and reduce social isolation, promoting better mental health outcomes through community and relational support.^25^ Finally, the engagement booster is essential for ensuring high participation and retention rates in the programme, thereby maximising the intervention’s overall impact.^26^ Although these three components are considered crucial, their effectiveness and cost-effectiveness remain uncertain, highlighting the need for thorough evaluations. Engagement boosters were found to increase the attendance of caregivers participating in the PLH for Young Children programme in three Eastern European countries (Foran *et al*), suggesting this component may also be relevant for adolescents and their caregivers’ engagement in the PLH for Parents and Teens programme.

This article presents the Phase 2 protocol of FLOURISH, which focuses on the optimisation of the adapted PLH programme by assessing the effectiveness and costs of HAT, adolescent peer support and the engagement boosters, as well as their combinations. By selecting the add-on components and combinations that prove most cost-effective, the PLH for Parents and Teens programme can be improved and more easily scaled up to maximise its impact on adolescent mental health, facilitating long-term delivery and sustainability in North Macedonia and Moldova. The optimal combination of add-on components in Phase 2 will be tested in a multicentre RCT in Phase 3.

The study is characterised by the following research questions.

1. What is the efficacy of each add-on component on primary and secondary outcomes?
2. What is the efficacy of the add-on components on implementation outcomes?
3. What are the costs of programme delivery and components?
4. What are the efficacy and cost consequences of the different combinations of components and what is the optimal combination to be tested in a multicentre RCT in Phase 3?
5. How is the programme implemented and received?

## Methods

### Study design

This study employs a multicountry cluster randomised factorial trial, according to the optimisation phase of the MOST framework. The study has been preregistered in clinical trials registry (NCT06562244) and follows the Consolidated Standards of Reporting Trials (CONSORT) guidelines.^27^ The standard protocol items: recommendations for interventional trials checklist is included as a supplementary document (online supplemental appendix 1).^28^

### Study setting

The study will be implemented in North Macedonia and Moldova and it is supported by two health networks: the Institute for Marriage, Family and Systemic Practice (ALTERNATIVA) in North Macedonia and the Health for Youth Association (HYA) in Moldova. ALTERNATIVA comprises a network of psychologists, social workers and family therapists. HYA supports publicly funded youth clinics that deliver preventive and therapeutic services, addressing areas, such as sexual and reproductive wellness, psychological well-being, substance misuse and prevention of violence. Both health networks have experience in delivering PLH programmes within the MOST framework (Foran *et al* and Heinrichs *et al*).^16^

### Participants and recruitment

The study will involve primary caregivers and their adolescents aged 10–14 years. For inclusion in the study, caregivers will have to meet the following criteria: they must be at least 18 years old at the baseline assessment, live with the adolescent in a household where the adolescent has slept at least four nights per week in the previous month, be able to speak at least one of the local languages (Romanian or Macedonian) and provide consent for both their own and their adolescent’s participation in the study by signing an informed consent. Adolescents must be between 10 and 14 years old at the baseline assessment, and both the caregiver and the teen must agree to participate in the study. In North Macedonia, adolescents aged≥14 are also required to provide consent in order to comply with local legal and ethical requirements. An example of the informed consent for caregivers and adolescents is included as a supplementary document (online supplemental appendix 2). Exclusion criteria for both caregivers and adolescents include significant psychiatric disorders, acute distress or physical health conditions that could impede their ability to participate in the study. These are assessed through an exclusionary statement as a part of the consent procedures.

All interested families meeting the criteria above will be eligible to participate in the study, with additional efforts made to include more economically and socially vulnerable groups using a proportionate universalism approach. This method focuses on individuals at higher than average risk without the need for preliminary screening.^29^ In North Macedonia, the recruitment process will primarily take place in schools, whereas in Moldova, it will be conducted in youth clinics. Families will be invited via multiple means to join the study (eg, school referrals, clinic referrals and community outreach) and provided an informational document that explains the study’s objectives, intervention specifics, the voluntary nature of involvement, data handling procedures, potential risks and benefits and provides contact details for additional questions or clarifications.

The programme will be implemented by the current staff and established networks of ALTERNATIVA and HYA. This staff includes data assessors, facilitators and supervisors. Data assessors are trained research staff responsible for administering participant assessments, ensuring the accurate data collection. Facilitators are trained professionals with prior experience in parenting interventions, responsible for delivering the intervention, leading sessions and guiding participants through skill-building activities. Supervisors are experienced senior staff who provide oversight and support to facilitators, ensure programme fidelity and conduct facilitator performance evaluations. Facilitators, supervisors and data assessors must be 18 years or older, participate in training, agree to fulfil their respective roles in programme delivery, supervision or assessment and provide consent.

### Intervention

To assess the efficacy and costs of the three add-on components (HAT, adolescent peer support and engagement booster) to the adapted PLH for Parents and Teens programme, the present study will implement a factorial cluster RCT.^22^

#### Intervention core programme: PLH for parents and teens programme

The core programme implemented in the FLOURISH project is PLH for Parents and Teens. In Phase 1 of FLOURISH, the PLH programme followed an adaptation drawing on the PLH manual used in a South African evaluation study and other programme versions.^23^ To support scalability, the PLH programme is condensed into six 2-hour weekly group sessions and delivered to groups of 10–12 adolescent–caregiver pairs. In each weekly session, a new skill relevant to adolescence is introduced and practised. Often, the group is split into parallel adolescent and caregiver sessions to allow open sharing. Home practice tasks are also assigned between sessions to reinforce learning. For example, after a session on building positive relationships, caregivers and adolescents are asked to spend at least 15 min of uninterrupted quality time together each day, during which the adolescent chooses an activity they enjoy. Caregivers are also encouraged to practice structured praise, offering at least one specific, positive comment to their adolescent each day. The programme is delivered by two facilitators per group with weekly supervision to ensure high-quality delivery. Facilitators, supervisors and coordinators complete trainings on the PLH programme and facilitation skills. Supervisors receive additional 2-day training on supervision techniques and on using the PLH facilitators assessment tool (PLH-FAT)^30^ to evaluate the facilitators’ performance in delivering the programme and provide supervision. For Phase 2, the core programme will maintain the same structure, with refinements based on the qualitative feedback, primarily from advisory and focus groups from Phase 1 (eg, less focus on sexuality, changes in the order of the topics as well as pilot feasibility study results; Shenderovich *et al*; Waller and Mueller *et al*). The adapted manual is freely available on the project website (https://www.flourish-study.org/).

#### Component 1: HAT (on/off)

The HAT component,^24^ which operates in an on/off mode (ie, delivered vs not delivered), consists of a group workshop designed to provide adolescents with essential skills using the UNICEFs ‘Magnificent Mei’ comic series and corresponding workbook for teenagers and facilitators.^31^ The workshop is delivered by a facilitator and includes two interactive sessions. Each session lasts for approximately 2 hours and focuses on four out of six selected chapters from the comics. The selected topics from the ‘Magnificent Mei’ comic series are: recognising emotions, dealing with stress, dealing with problems and working out conflict and being there for each other. In ‘recognising emotions’, adolescents learn to identify and express their feelings. ‘Dealing with stress’ teaches them about the effects of stress on the body and calming techniques like breathing exercises. ‘Dealing with problems’ covers conflict resolution and effective communication among friends and family. ‘Working out conflict and being there for each other’ emphasises listening and understanding different perspectives, concluding with a lesson on building strong relationships through mutual support. In Phase 2 of the FLOURISH project, the workshop will be implemented between the third and fifth PLH sessions (weeks 4 and 6).

#### Component 2: adolescent peer support (on/off)

The adolescent peer support component is designed to teach adolescents how to support friends in distress. Based on UNICEFs ‘I Support My Friends’ initiative^32^ and adaptions for the FLOURISH project, the programme consists of a half-day workshop divided into three main parts: look, listen and link. In the ‘look’ part, adolescents learn to identify signs of distress in peers. The ‘listen’ part focuses on developing active listening skills, while the ‘link’ part teaches when and how to seek additional support. As a result, adolescents receiving this component may acquire essential skills to recognise signs of distress in their peers and offer immediate support. They will learn to refer friends to adults for further help, respond appropriately to their friends’ emotional reactions, assist in accessing necessary resources and services and ensure their friends’ safety from further harm. Furthermore, by emphasising the importance of seeking help and support in challenging situations, the programme equips children and adolescents to assist their peers effectively. The workshop targets groups of 10–12 adolescents and will be delivered following the first PLH session (week 2).

#### Component 3: engagement booster (high/low)

We will include and evaluate a specific component designed to motivate families to commit to and complete the PLH programme. This component will consist of two levels of incentives. In the low level, all families receive minimal refreshments at each session. Additionally, all families receive a certificate of attendance if they attend one to four out of six PLH sessions or a certificate of successful completion if they attend five or six sessions. In the low level, all families receive refreshments at each session. In the high-level condition, participants additionally receive vouchers as incentives. Youth receive a voucher (equivalent to €10) for attending five or six sessions, and caregivers receive a voucher (equivalent to €5) for the same level of attendance. Both adolescents and parents can attend the sessions independently of each other, and the incentives are based on their individual attendance. Certificates and vouchers will be distributed during a small closing ceremony at the end of the sixth PLH session (week 7).

Table 1 shows the adapted intervention structure, including the PLH core programme and the three components.

**Table 1:** Adapted intervention structure for phase 2 optimisation study

Week	Session	Core programme	Components		
		PLH for parents and teens*	HAT†	Engagement booster	Adolescent peer support†
1	0	Pregroup session home visits		Refreshments*	
2	1	Developmental stages‡		Refreshments*	Look, listen, link workshop
3	2	Building a positive relationship through spending time together§		Refreshments*	
4	3	Talking about emotions and sensitive topics‡	Recognising emotions and dealing with stress	Refreshments*	
5	4	What do we do when we are angry‡		Refreshments*	
6	5	Establishing rules and routines§	Dealing with problems and working out conflict and being there for each other	Refreshments*	
7	6	Problem solving‡		Refreshments* Certificate of attendance* Certificate of successful completion (five–six PLH sessions)* €10 voucher for adolescents and €5 for caregivers (five–six PLH sessions each)¶	

* Delivered to all families.

† Delivered to adolescents only in specific experimental conditions.

‡ Caregivers and adolescents’ joint and separate session.

§ Caregivers and adolescents’ joint session.

¶ High-level component: only in specific experimental conditions.

HAT, helping adolescents thrive; PLH, parenting for lifelong health.

### Experimental conditions

The factorial experiment will consist of eight conditions (2^3^), stratified by country (table 2).

**Table 2:** Experimental conditions

Condition number	Components		
	HAT	Engagement booster	Adolescent peer support
1	On	High	On
2	On	High	Off
3	On	Low	On
4	On	Low	Off
5	Off	High	On
6	Off	High	Off
7	Off	Low	On
8	Off	Low	Off

Note. On/Off=delivered/not delivered.

HAT, helping adolescents thrive.

### Hypotheses of the study

**Figure 1:**
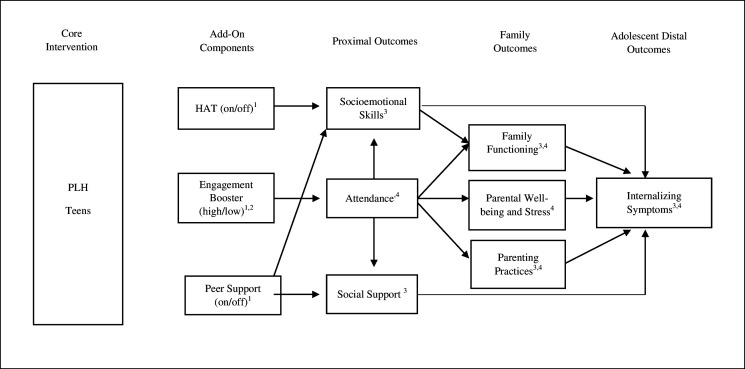
Conceptual model for PLH Teens and add-on components. Notes: ^1^Delivered to adolescents; ^2^Delivered to caregivers; ^3^Reported by adolescents; ^4^ Reported by caregivers. Additional direct paths and interaction effects between components are not included in the figure for simplicity. We hypothesise that caregivers and adolescents across all conditions will benefit from the core PLH programme, resulting in improvements in family communication and positive parenting, as well as a reduction in parental distress. These changes are expected to be associated with enhancements in adolescent mental health and well-being. HAT, helping adolescents thrive; PLH, parenting for lifelong health.

Figure 1 presents the conceptual model for the current study, including the theory of change regarding the PLH for Parents and Teens programme and the components effects. Each hypothesis is directly connected to specific outcomes, in line with causal models for RCTs of complex interventions.^33^

It is hypothesised the following.

1. Caregivers and adolescents across all conditions will benefit from the adapted core PLH for Parents and Teens programme, resulting in improvements in primary and secondary outcomes (eg, family communication and parenting, as well as a reduction in parental stress). These changes are expected to be associated with improvements in adolescent mental health (direct and indirect effects).
2. Adolescents receiving the HAT component will show better socioemotional skills compared with those not receiving the HAT component. This, in turn, will lead to greater improvements in family functioning (eg, family communication) and adolescent mental health.
3. Caregivers and adolescents participating in the high engagement component will exhibit higher attendance rates in PLH compared with those involved in the low engagement component. This higher rate of participation is anticipated to lead to greater improvements in both primary (eg, adolescent mental health, via indirect effects and social support) and secondary outcomes (eg, family functioning, parenting and parental stress).
4. Adolescents receiving the peer support component will show greater improvements in social support and socioemotional skills compared with those not receiving the peer component. This, in turn, will lead to greater improvements in family functioning and adolescent mental health.

Additional exploratory hypotheses.

1. There will be a significant interaction between the engagement booster component and the adolescent peer support component. Specifically, when both components are high/on, there will be greater improvements in primary outcomes (eg, adolescent mental health and social support) compared with when either component is low/off. These improvements are expected to occur through indirect effects, such as enhanced socioemotional skills and family communication.
2. The combination of the HAT component and adolescent peer support component will result in significantly greater improvements in both primary (eg, adolescent mental health) and secondary outcomes (eg, family functioning) when both components are on compared with when either component is off (ie, interaction effect). These improvements are expected to occur through indirect effects, such as enhanced socioemotional skills and increased social support.
3. The HAT component and engagement booster component will interact to produce greater improvements in adolescent mental health, participation and socioemotional skills when both components are high/on compared with when either component is low/off.
4. The PLH programme will be implemented with high fidelity and will be well received by participants, with feedback from participants indicating high satisfaction.
5. The programme will have broader cost consequences beyond direct implementation costs, and different component combinations will have different incremental efficacies and costs representing alternative optimisation scenarios in terms of cost-effectiveness.

### Sample size

A power analysis was conducted to determine the sample size required for this study, using the ‘FactorialPowerPlan’ function within the R-package ‘MOST’.^34^ The study is designed as a factorial experiment with 3 factors and 64 clusters (eight conditions with four groups per condition per country). To detect a small effect with an 80% power and alpha of 0.10 (as recommended for factorial trials in MOST),^35^ considering a small amount of intraclass correlation (ICC) in the absence of intervention (ie, ICC=0.01) and low clustering of change scores (ICC of change scores=0.10), the sample size should include approximately 7 families per cluster, corresponding to 448 families. To consider drop-out after recruitment, it is estimated to recruit approximately 10–12 families per cluster (640–768 families).

### Randomisation and blinding

The randomisation will be carried out at the University of Klagenfurt. A randomisation website (https://www.randomizer.org) will be used to generate random numbers for allocating clusters to different conditions. Randomisation will be stratified by country, with four clusters from each country assigned to one of the eight experimental conditions. The randomisation list will be shared with the research manager at ALTERNATIVA and HYA. This information will be shared via email from H.M. Foran (University of Klagenfurt) to the researcher managers at ALTERNATIVA and HYA only. They will keep this information concealed from all participants until after baseline assessments.

At the baseline assessment, both participants and data assessors will be unaware of the group assignments. After the baseline data collection is finished, the research managers at each site will notify the participating families of their group allocation. Data assessors responsible for the assessments will also be blinded to the allocation throughout the study including at postassessment to minimise bias. However, after baseline assessments, participants cannot remain blind due to their involvement in the programme implementation. To maintain the integrity of the study, contamination will be rigorously monitored.

### Data collection and outcomes

Outcomes will be assessed at baseline and postassessment, approximately, 8 weeks later. Data collection from adolescents and caregivers will be conducted primarily using computer-assisted self-report questionnaires on tablets (open-source software platform, Open Data Kit), with data assessors assisting adolescents and caregivers in completing the questionnaires. Adolescents and caregivers will complete assessments separately. Data assessors will receive comprehensive training from the study investigators. This training will cover ethics, informed consent procedures, safety procedures, adverse event reporting and interviewing techniques.

Outcome measures without official translations were adapted and translated following best practice guidelines for cross-cultural adaptation (Piolanti *et al*; Waller and Mueller *et al*).

#### Primary outcomes

The study will assess four primary outcomes: adolescent emotional problems (internalising), adolescent social support, family functioning and attendance (for both adolescents and caregivers). Adolescent internalising problems will be assessed using the parent-report version of the child behaviour checklist (CBCL) for ages 6–18.^36^ Adolescent social support will be measured using the adolescent-report Kidscreen-52, peers and social support subscale^37^ and the medical outcome study social support survey, emotional support subscale.^38^ Family functioning will be assessed using the family assessment device, general family functioning subscale, as reported by caregivers.^39^ Attendance will be measured with the percentage of group PLH sessions attended by caregivers and adolescents.

#### Secondary outcomes

Secondary outcomes encompass additional endpoints expected to show changes as a result of the PLH programme and add-on components. These include adolescent- and caregiver-reported measures of family communication and functioning, parenting practices, loneliness, social support, well-being, behavioural and emotional problems, psychological distress, parental stress, socioemotional skills and health-economic measures. The full list of questionnaires for these and subsequent outcomes is provided in the supplementary material (online supplemental table S1 and S2, appendix 3). Concerning the health-economic evaluation, tools will include standardised measures of health-related quality of life, such as the EQ-5D-5L for caregivers and EQ-5D-Y-3L for adolescents.^40^ In addition, the economic evaluation will incorporate two specific tools: the Oxford CAPabilities questionnaire—Mental Health^41^ completed by caregivers, which provides a multidimensional capability well-being assessment, and version 2 of the PECUNIA resource use measurement instrument,^42^ for caregivers and a proxy version for adolescents, which provides a detailed assessment of broader resource use and will be costed using internationally validated unit costing methods.^43^

#### Other prespecified outcomes

Other prespecified outcomes include measures related to participant characteristics, costs as well as implementation and staff outcomes that may influence the programme’s effectiveness. These outcomes include adolescent body mass index, assessed for descriptive purposes using self-reported and caregiver-reported height and weight; caregiver alcohol use; adolescent-defined problems, where adolescents identify and rate the severity of their top concerns; and post-traumatic stress in both adolescents and caregivers (online supplemental table S2, appendix 3).

Direct implementation cost data will be collected from multiple sources. A purposefully designed cost questionnaire will capture the comprehensive personnel time associated with the training and delivery of the intervention, while additional organisational, material, transportation and consumable costs will be gathered from finance records.

Implementation and staff outcomes include the collection of enrolment rates, weekly attendance records by facilitators and fidelity assessments of PLH sessions conducted by programme supervisors. The implementation of the HAT component and the adolescent peer components will be assessed using a short questionnaire filled out by the facilitators. PLH sessions in both countries will be recorded for supervisory purposes, with one session being assessed using the PLH-FAT.^30^ Additionally, facilitators and supervisors will complete questionnaires on well-being and, for those who are caregivers, parental stress.

#### Process evaluation

Postintervention focus groups will be organised with a subset of adolescents, caregivers and intervention staff. Each focus group will consist of six to eight participants, with a minimum of four groups conducted in each country (adolescents, parents, facilitators and supervisors). The purpose of these focus groups is to collect information for further programme adaptations and to address process evaluation questions regarding the context, implementation and mechanisms of change of the programme. We will also seek to capture feedback on various programme component combinations by including participants who experienced all programme components (all on/high condition).

### Statistical analysis

Analyses will examine the main effect of each component and interaction effects using regression analyses, with adjustments based on baseline data. For pre- and postassessments, intent-to-treat analyses accounting for clustering and stratification by country will be used. Separate models will be tested using effect coding for treatment effects.^44^

Indirect models based on the hypothesised theory of change will be examined using a Mplus software^45^ or R^46^ and accounting for clustering within groups and using bootstrapping procedures (eg, engagement booster component increasing attendance, which, in turn, is related to greater changes in primary and secondary outcomes). Multigroup analyses or t-tests at baseline will be performed to identify differences between countries and other contextual or demographic variables. Any missing data will be assessed and managed using either multiple imputation or full information maximum likelihood estimation, following the intention-to-treat analysis protocol.^47^

#### Health-economics analysis

To comprehensively evaluate the value of the eight experimental combinations, we will employ a cost-consequences analysis (CCA).^48^ The CCA examines the cost and outcomes for each experimental combination, detailing the cost of resources used and the (health) outcomes achieved (eg, changes in CBCL internalising scores^36^ and EQ-5D-Y scores).^40^ This information allows for a clear comparison of the relative value of each combination by presenting both cost and outcome impacts alongside each other systematically and provides the basis for the planned optimisation strategy using a multicriteria decision analysis (MCDA) framework.^49^

#### Process evaluation and staff outcomes analysis

Descriptive statistics will be employed to summarise data concerning attendance, fidelity and characteristics of facilitators. Additionally, the study will explore differences in attendance linked to the baseline characteristics of participants (eg, gender) and characteristics of the intervention’s delivery (eg, study location), as well as changes in staff well-being based on the questionnaires administered before and after the intervention.

### Qualitative analysis

Transcripts from the focus groups will be analysed using thematic and framework analysis,^50^ drawing on both inductive and deductive approaches to generate codes.

### Selection of the optimised intervention: decision process

The criteria for optimisation in this study are designed to identify and include the most beneficial and good value for money, scalable combination for the upcoming RCT in Phase 3 of FLOURISH. To achieve this, we will implement a systematic selection process. We will first assess the effect of each component level (ie, different levels of administration vs non-administration) on study outcomes. Next, we will examine two-way and three-way interactions between the components. The various component combinations will be compared based on the incremental increases in overall benefit and overall cost within an MCDA. Those options that are dominated will be eliminated. For the remaining options, we will use cost–benefit optimisation to select the final combination for Phase 3.

### Patient and public involvement

Advisory groups were set up and consulted on the existing services and challenges, and intervention design and scaling-up considerations in each country, informing the design of the programme tested in Phase 1. The groups include adolescents, caregivers, intervention staff and professional experts (six to eight participants and four groups in each country). The advisory groups are planned to be consulted for further feedback during the project. Any feedback would be summarised in notes, organised into a matrix by key themes and stakeholder type and analysed using thematic content analysis.

### Participant timeline

The study implementation timeline varies between North Macedonia and Moldova to accommodate practical considerations unique to each context. Figure S1 in the online supplemental material, appendix 3 shows the study flowchart. Recruitment of potential caregivers and adolescents will begin after cluster randomisation and is scheduled for October 2024 in North Macedonia and December 2024 in Moldova. Recruitment will continue until the target sample size of 320 enrolled families per country is achieved. In North Macedonia, the intervention and related assessments will be conducted in two waves (beginning in October 2024 and January 2025), while one wave will be used in Moldova (beginning in January 2025). Specific dates may be subject to change to accommodate any practical deviations that arise during the study implementation. These timing variations will be considered in the analyses. Randomisation will ensure that each phase receives all eight conditions, maintaining balance.

## Ethics and dissemination

All participants will be clearly informed of their right to decline participation or withdraw from the study at any time without affecting their access to other services.

Staff members will be trained to identify and address adverse events caused by the research or intervention, guided by an adverse event and safety protocol. In case of disclosure of self-harm by a participant, staff will provide immediate risk assessment, support and referral. All serious adverse events will be reported to the local Principal investigator (PI), the study coordinator at the University of Klagenfurt, the responsible ethics boards and the data safety and monitoring board (DSMB) for review. The DSMB and ethical boards will independently review the events to decide if any changes to the project are needed. Support is also available for the research team, with guidance provided on how to manage distressing disclosures and access emotional support if required. This is also a part of the trainings for project personnel involved in the study.

Data management will adhere to the recommendations of good clinical practice, the Declaration of Helsinki and CONSORT guidelines. Non-electronic data will be securely stored in locked cabinets at the institution responsible for data collection. In line with ethical research conduct guidelines, these data will be retained for 15 years. After this period, the responsible researcher will oversee the destruction of the data and document the storage and deletion procedures. Electronic personal data collected for participant contact and session monitoring during the intervention will be stored in a password-protected file on password-protected computers and/or external hard drives in a locked room at the local country offices in North Macedonia and Moldova. All PLH sessions will be video recorded for facilitator supervision and quality assessment, as well as training. The video files will not be shared with anyone outside the country team and the ALTERNATIVA/HYA networks. Only selected members of the local FLOURISH team will have access to this data. All personal data will be kept confidential and will not be shared beyond the local research teams in North Macedonia and Moldova. Data collected via electronic tablets for assessments will be managed by the team at the University of Klagenfurt, which oversees the overall data management. These data will be transmitted electronically using ODK software to the server at the University of Klagenfurt, where no access to personal information that could identify participants will be available. The anonymised project data will be backed up on two additional password-protected servers and external hard drives managed by the IT department at the University of Klagenfurt.

Data oversight will be conducted by the Data Protection Officer (DPO), the DSMB and the Ethical Advisor. The DPO will be responsible for continuously reviewing the data management plan and protection measures to ensure that they comply with GDPR regulations. Any data protection breaches will be recorded and reported to the DPO. The DSMB, consisting of two experts with extensive experience in complex research involving families and children, will review study procedures, participant safety data, study conduct and progress. They will advise on whether the project should continue, be stopped or need adjustments.

Ethical approval for this study has been obtained from several bodies: the Institutional Review Board for Research Ethics at the University of Klagenfurt in Austria (approval number: 2023–013), the Ethical Commission for Human Research at the Medical Faculty of St. Cyril and Methodius University in North Macedonia (approval number: 03-2144/4) and the National Committee of Ethical Expertise for Clinical Trials at the Ministry of Health in Moldova (approval number: 1476).

The FLOURISH project is dedicated to sharing its findings with relevant stakeholders to ensure the broadest impact and application of the research and has a dissemination and exploitation plan that was prepared at the onset of the project and updated at regular intervals as a deliverable to the European Commission. Dissemination efforts will be multifaceted, encompassing a variety of strategies to reach a wide audience. These efforts will include organising meetings with key stakeholders, engaging with media outlets and distributing information through scientific publications for researchers and other summaries for various stakeholder groups. The project will leverage the existing networks, the FLOURISH project website and social media outlets to expand its reach and engagement.

The primary focus of dissemination activities will be in North Macedonia and Moldova, the countries where the research is being conducted. However, the project also aims to engage stakeholders across the broader Eastern European region. By working with these diverse partners, the project aims to promote that the research findings are integrated into policy and practice, ultimately improving adolescent mental health and well-being

This factorial study is designed to evaluate the effectiveness and costs of the adapted PLH for Parents and Teens programme and different add-on components, as well as their combination, to provide valuable insights that will inform the design of the subsequent RCT planned as the next phase of the FLOURISH project. This thorough methodology will support the FLOURISH project in creating scalable, evidence-based interventions designed to enhance the mental health and well-being of adolescents and their families in North Macedonia and Moldova.

## Supplementary material

10.1136/bmjopen-2024-094085 online supplemental file 1

10.1136/bmjopen-2024-094085 online supplemental file 2

10.1136/bmjopen-2024-094085 online supplemental file 3
