# Supplementary material for: Family-focused intervention programme to foster adolescent mental health and well-being: protocol for a multicountry cluster randomised factorial trial (FLOURISH Phase 2)
Source: BMJ Open. 2025 Feb 7;15(2):e094085. doi: 10.1136/bmjopen-2024-094085 (PMC11808879; doi:10.1136/bmjopen-2024-094085)
Supplement: online supplemental file 3 [file bmjopen-15-2-s003.docx]

**Supplementary Material: Appendix 3**

**Table S1**

*Secondary Outcome Measures*

| Construct | Respondent | Measure |
| --- | --- | --- |
| Adolescent emotional problems | Adolescents | The Revised Child Anxiety and Depression Scales (RCADS) (Ebesutani et al., 2012) |
|  | Adolescents & caregivers | Pediatric Symptom Checklist (PSC) (Jellinek et al., 1986) |
| Adolescent behavior problems | Caregivers | Child Behavior Checklist 6-18, externalizing behavior (Achenbach & Rescorla, 2001) |
| Family communication | Adolescents | Child-Parent Communication Apprehension scale (Lucchetti et al., 2002) |
| Family functioning | Caregivers | Family Assessment Device-FAD, problem solving and communication subscales (Epstein et al., 1983) |
| Parenting practices (positive parenting) | Adolescents & caregivers | Alabama Parenting Questionnaire (Frick, 1991), involved parenting and positive parenting subscales. |
| Parenting practices (harsh parenting) | Caregivers | Alabama Parenting Questionnaire (Frick, 1991), corporal punishment subscale. |
| Loneliness | Adolescents | UCLA-8 Loneliness scale (Roberts et al., 1993) |
|  | Caregivers | Revised UCLA-6 Loneliness scale (Wongpakaran et al., 2020) |
| Social support | Adolescents &  Caregivers | Medical Outcome Study Social Support Survey, emotional and affectionate sub-scales (Sherbourne & Stewart, 1991). |
| Wellbeing | Adolescents & caregivers | WHO-5 Well-Being Index (WHO-5; Topp et al., 2015). |
| Socio-emotional skills | Adolescents | Social Emotional Abilities and Learning (SEAL) Tool (UNICEF, n.d.) |
| Caregiver psychological distress | Caregivers | The Patient Health Questionnaire – 9 (PHQ-9) (Kroenke et al., 2001). |
| Parental stress | Caregivers | Parental Stress Scale (Berry & Jones, 1995). |
| Health-related quality of life | Adolescents | EQ-5D-Y-3L (EuroQol Research Foundation, 2024). |
|  | Caregivers | EQ-5D-5L (Herdman et al., 2011). |
| Caregiver capabilities | Caregivers | Oxford CAPabilities questionnaire - Mental Health (OxCAP-MH) (Simon et al., 2013). |
| Caregiver resource use | Caregivers | PECUNIA Resource Use Measurement (PECUNIA RUM) (Pokhilenko et al., 2023). |
| Caregiver-reported resource use in adolescents | Caregivers | PECUNIA Resource Use Measurement (PECUNIA RUM) proxy for adolescents. |

**Table S2**

*Other Pre-Specified Measures*

| Construct | Respondent | Measure |
| --- | --- | --- |
| Adolescent-defined problems | Adolescents | The Top Problem Assessment (Milgram et al., 2021; Weisz, 2012; Weisz et al., 2011). |
| Alcohol use | Caregivers | The Alcohol Use Disorder Identification Test (Saunders et al., 1993). |
| Posttraumatic stress | Caregivers | PTSD checklist for DSM-5 (PCL-5) (Weathers et al., 2013) |
|  | Adolescents | Children’s Revised Impact of Event Scale (Perrin et al., 2005) |
| Wellbeing | Intervention Staff | WHO-5 Well-Being Index (WHO-5; Topp et al., 2015). |
| Parental Stress | Intervention Staff | Parental Stress Scale (Berry & Jones, 1995). |
| Adolescent healthy weight | Adolescents & Caregivers | BMI (caregiver and adolescent self-report on mass in kilograms and height in meters). |
| Fidelity of intervention delivery | Facilitators | PLH-Facilitator Assessment Tool (PLH-FAT) (Martin et al., 2023). |
| Attendance rate | Intervention Staff | Percentage of group PLH sessions attended by caregivers and adolescents; Percentage of catch-up PLH contact; Percentage of adolescent participation in Magnificent Mei and I Support My Friends. |
| Enrollment rate | Intervention Staff | Percentage of participants who attended at least one PLH session (adolescents, caregivers, and families). |
| Intervention costs | Intervention Staff | Intervention costs: Personnel time, material, transportation, consumables, and organizational costs. |

**REFERENCES**

Achenbach, T. M., & Rescorla, L. (2001). *The Manual for the ASEBA School-Age Forms & Profiles.*

Berry, J. O., & Jones, W. H. (1995). The parental stress scale: Initial psychometric evidence. *Journal of Social and Personal Relationships*, *12*(3), 463–472. https://doi.org/10.1177/0265407595123009

Ebesutani, C., Reise, S., Chorpita, B. F., Ale, C., Regan, J., Young, J., Higa-McMillan, C., & Weisz, J. (2012). The Revised Child Anxiety and Depression Scale - Short Version: Scale reduction via exploratory bifactor modeling of the broad anxiety factor. *Psychological Assessment, 24*, 833-845.

Epstein, N. B., Baldwin, L. M., & Bishop, D. S. (1983). The McMaster family assessment device. *Journal of Marital and Family Therapy*, *9*(2), 171–180. https://doi.org/10.1111/j.1752-0606.1983.tb01497.x

EuroQol Research Foundation. (2024). EQ-5D-Y-3L user guide (Version 2.1). EuroQol Research Foundation.

Frick, P. J. (1991). Alabama Parenting Questionnaire. *Unpublished Rating Scale, University of Alabama.*

Herdman, M., Gudex, C., Lloyd, A., Janssen, M. F., Kind, P., Parkin, D., Bonsel, G., & Badia, X. (2011). Development and preliminary testing of the new five-level version of EQ-5D (EQ-5D-5L). Quality of Life Research, 20(10), 1727–1736. https://doi.org/10.1007/s11136-011-9903-x

Krause, K. R., Chung, S., Adewuya, A. O., Albano, A. M., Babins-Wagner, R., Birkinshaw, L., Brann, P., Creswell, C., Delaney, K., Falissard, B., Forrest, C. B., Hudson, J. L., Ishikawa, S., Khatwani, M., Kieling, C., Krause, J., Malik, K., Martínez, V., Mughal, F., … Wolpert, M. (2021). International consensus on a standard set of outcome measures for child and youth anxiety, depression, obsessive-compulsive disorder, and post-traumatic stress disorder. *The Lancet Psychiatry*, *8*(1), 76–86. https://doi.org/10.1016/S2215-0366(20)30356-4

Kroenke, K., Spitzer, R. L., & Williams, J. B. W. (2001). The PHQ-9: validity of a brief depression severity measure. *Journal of General Internal Medicine*, *16*(9), 606–613. https://doi.org/10.1046/j.1525-1497.2001.016009606.x

Jellinek, M. S., Murphy, J. M., Robinson, J., Feins, A., Lamb, S., & Fenton, T. (1986). Pediatric Symptom Checklist: Screening school-age children for psychosocial dysfunction. Journal of Pediatrics, 109(2), 258-264.

Lucchetti, A. E., Powers, W. G., & Love, D. E. (2002). The Empirical Development of the Child-Parent Communication Apprehension Scale for Use With Young Adults. *Journal of Family Communication*, *2*(3), 109–131. https://doi.org/10.1207/S15327698JFC0203_1

Martin, M., Lachman, J. M., Murphy, H., Ward, C. L., Hutchings, J., Cluver, L., & Gardner, F. (2023). The development, reliability, and validity of the Facilitator Assessment Tool: An implementation fidelity measure used in Parenting for Lifelong Health for Young Children. Child: Care, Health and Development, 49(5), 591–604.

Milgram, L., Tonarely, N. A., & Ehrenreich-May, J. (2021). Youth top problems and early treatment response to the unified protocols for transdiagnostic treatment of emotional disorders in children and adolescents. *Child Psychiatry & Human Development*. https://doi.org/10.1007/s10578-021-01151-4

Perrin, S., Meiser-Stedman, R., & Smith, P. (2005). The Children’s Revised Impact of Event Scale (CRIES): Validity as a Screening Instrument for PTSD. *Behavioural and Cognitive Psychotherapy*, *33*(4), 487–498. https://doi.org/10.1017/S1352465805002419

Pokhilenko, I., Janssen, L. M. M., Paulus, A. T. G., van Ginneken, E., & Busse, R. (2023). Development of an instrument for the assessment of health-related multi-sectoral resource use in Europe: The PECUNIA RUM. Applied Health Economics and Health Policy, 21(2), 155–166. https://doi.org/10.1007/s40258-022-00780-7

Ravens-Sieberer, U., Auquier, P., Erhart, M., Gosch, A., Rajmil, L., Bruil, J., ... & Czemy, L. (2007). The KIDSCREEN-52 quality of life measure for children and adolescents: psychometric results from a cross- cultural survey in 13 European countries. Value in Health, 11(4), 645-658.

Roberts, R. E., Lewinsohn, P. M., & Seeley, J. R. (1993). A Brief Measure of Loneliness Suitable for Use with Adolescents. *Psychological Reports*, *72*(3_suppl), 1379–1391. https://doi.org/10.2466/pr0.1993.72.3c.1379

Russell, D. W. (1996). UCLA Loneliness Scale (Version 3): Reliability, Validity, and Factor Structure. *Journal of Personality Assessment*, *66*(1), 20–40. https://doi.org/10.1207/s15327752jpa6601_2

Saunders, J. B., Aasland, O. G., Babor, T. F., De la Fuente, J. R., & Grant, M. (1993). Development of the Alcohol Use Disorders Identification Test (AUDIT): WHO Collaborative Project on Early Detection of Persons with Harmful Alcohol Consumption‐II. *Addiction*, *88*(6), 791–804. https://doi.org/10.1111/j.1360-0443.1993.tb02093.x

Sherbourne, C. D., & Stewart, A. L. (1991). The MOS social support survey. *Social Science & Medicine, 32*(6), 705-714. https://doi.org/10.1016/0277-9536(91)90150-B

Simon, J., Anand, P., Gray, A., Rugkåsa, J., Yeeles, K., & Burns, T. (2013). Operationalising the capability approach for outcome measurement in mental health research. Social Science & Medicine, 98, 187–196. https://doi.org/10.1016/j.socscimed.2013.09.019

Topp, C. W., Østergaard, S. D., Søndergaard, S., & Bech, P. (2015). The WHO-5 Well-Being Index: A systematic review of the literature. Psychotherapy and Psychosomatics, 84(3), 167-176.

UNICEF. (n.d.). *SEA baseline-endline Tool*. Retrieved from https://docs.google.com/forms/d/e/1FAIpQLSfsAm25WPFw0-oJip_P-LjVfwNhXMOo2C_cIjUIeqQqn6szGQ/viewform

Weathers, F. W., Litz, B. T., Keane, T. M., Palmieri, P. A., Marx, B. P., & Schnurr, P. P. (2013). The PTSD Checklist for DSM-5 (PCL-5). National Center for PTSD.

Weisz, J. R. (2012). Testing standard and modular designs for psychotherapy treating depression, anxiety, and conduct problems in youth. *Archives of General Psychiatry*, *69*(3), 274. https://doi.org/10.1001/archgenpsychiatry.2011.147

Weisz, J. R., Chorpita, B. F., Frye, A., Ng, M. Y., Lau, N., Bearman, S. K., Ugueto, A. M., Langer, D. A., & Hoagwood, K. E. (2011). Youth top problems: Using idiographic, consumer-guided assessment to identify treatment needs and to track change during psychotherapy. *Journal of Consulting and Clinical Psychology*, *79*(3), 369–380. https://doi.org/10.1037/a0023307

Wongpakaran, N., Wongpakaran, T., Pinyopornpanish, M., Simcharoen, S., Suradom, C., Varnado, P., & Kuntawong, P. (2020). Development and validation of a 6‐item Revised UCLA Loneliness Scale (RULS‐6) using Rasch analysis. *British Journal of Health Psychology*, *25*(2), 233–256. https://doi.org/10.1111/bjhp.12404

**Figure S1.**

*Study Flow Chart*

Post-intervention assessment

Intervention implementation (7 weeks)

Baseline assessment

Recruitment of 10-12 families per cluster (caregiver + adolescent aged 10-14 years)

Recruitment of 64 clusters

(32 per country)

Randomization of clusters to 8 experimental conditions:

- 4 clusters per condition per country

- Each country has the same 8 conditions
