# Supplementary material for: Family-focused intervention programme to foster adolescent mental health and well-being: protocol for a multicountry cluster randomised factorial trial (FLOURISH Phase 2)
Source: BMJ Open. 2025 Feb 7;15(2):e094085. doi: 10.1136/bmjopen-2024-094085 (PMC11808879; doi:10.1136/bmjopen-2024-094085)
Supplement: Supplementary file 4 [file bmjopen-2024-094085.reviewer_comments.pdf]

PEER REVIEW HISTORY

BMJ Open publishes all reviews undertaken for accepted manuscripts. Reviewers are asked to complete a checklist review form and are provided with free text boxes to elaborate on their assessment. These free text comments are reproduced below.

ARTICLE DETAILS

Title (Provisional)

A Family-Focused Intervention Program to Foster Adolescent Mental Health and Well-Being: Protocol for a Multi-Country Cluster Randomized Factorial Trial (FLOURISH Phase 2)

Authors

Piolanti, Antonio; Mueller, Janina; Waller, Franziska; Heinrichs, Nina; Simon, Judith; Shenderovich, Yulia; Sampathkumar, Swetha; Wienand, Dennis; Raleva, Marija; Kunovski, Ivo; Babii, Viorel; Zhao, Xiang; Moore, Graham; Foran, Heather M.

VERSION 1 - REVIEW

**Reviewer**	**1**
**Name**	**Cleverley, Kristin**
**Affiliation**	**University of Toronto, Nursing**
**Date**	**13-Nov-2024**
**COI**	**I understand and consent to the named publication of this review.**

Thank you for the opportunity to review this protocol. It was a well written protocol, easy to follow, and followed the SPIRIT guidelines. A few minor comments/suggestions to the authors.

Page 8 (participants and recruitment), lines 11-13. Please provide further details on how “presence of severe psychological problems or an acute mental disability” will be both defined and assessed.

Page 8, line 21, what does “invited via multiple means” refer too?

Page 8, lines 18-20, will type of recruitment site be evaluated in the study? It is not overviewed in methods/analysis. However, these are very different settings. “In North Macedonia, the recruitment process will primarily take place in schools, whereas in Moldova, it will be conducted in youth clinics.” Also, please define “youth clinics”.

Page 13, lines 9 (primary outcomes). How were the primary outcomes selected? Why only parent/caregiver reported mental health outcome (CBCL)? Would a self-report assessment of mental health/functioning be warranted?

How will potential risk (ie disclosure of suicidal thoughts/ideation) be handled during the study? Is there a protocol in place? Will there be clinical support available to the research teams?

Page14, statistical analysis section; please add appropriate citations throughout section (there are none included); including the quantitative, health economics, and qualitative sections.

**Reviewer**	**2**
**Name**	**Doohan, Meg**
**Affiliation**	**Griffith University - Nathan Campus**
**Date**	**29-Nov-2024**
**COI review.**	**I understand and consent to the named publication of this**

Abstract: Line 19 - Can the term 'engagement booster' be better explained or elaborated on?

Throughout Manuscript: Please ensure the use of numerals <10 (unless applied to a specific phase) are written as words, consistently, throughout the manuscript.

Methods: Page 7 - The role of and training required for supervisors in the program is unclear. Who are these people, what supervision are they agreeing to provide, and what training will they be participating in?

Methods: Page 7, Line 45 - Provide an example of home practice tasks to be assigned between sessions.

Methods - Experimental Conditions: A diagram of the stratification of experimental conditions between countries would be worthwhile for clarity and understanding of the methodology.

Participant Timeline - Page 14: Please update the timeline to reflect the most likely data collection period (i.e., not starting late 2024).

Ethics and Dissemination - Page 14: Please clarify whether adolescents will be providing consent themselves to engage in the programs, or if this consent will be provided by parents/caregivers.

VERSION 1 - AUTHOR RESPONSE

Reviewer #1

- Page 8 (participants and recruitment), lines 11-13. Please provide further details on how "presence of severe psychological problems or an acute mental disability" will be both defined and assessed.

Response: Thank you for your feedback. The exclusion criteria for "severe psychological problems or an acute mental disability" are evaluated during the consent procedures. This process includes an exclusionary statement indicating that the program is not suitable for individuals experiencing acute distress or significant psychiatric disorders that could impede their ability to participate effectively. Participants who indicate these concerns are referred to alternative services. We have revised the sentence in the manuscript: "Exclusion criteria for both caregivers and adolescents include significant psychiatric disorders, acute distress, or physical health conditions that could impede their ability to participate in the study. These are assessed through an exclusionary statement as part of the consent procedures." Data assessors are also trained in safety procedures in the cases that a participant shows visible signs of distress or other psychological problems or impairment. In these cases, the data assessor will involve the supervisor and provide referrals for additional services as appropriate. Both settings are connected to existing service organizations where referrals services can be provided.

-Page 8, line 21, what does "invited via multiple means" refer too?

Response: Thank you for the suggestion to clarify this point. We have modified the sentence as follows: "Families will be invited via multiple means to join the study (e.g., school referrals, clinic referrals, and community outreach) and provided an informational document that explains the study's objectives, intervention specifics, the voluntary nature of involvement, data handling procedures, potential risks and benefits, and provides contact

details for additional questions or clarifications."

- Page 8, lines 18-20, will type of recruitment site be evaluated in the study? It is not overviewed in methods/analysis. However, these are very different settings. "In North Macedonia, the recruitment process will primarily take place in schools, whereas in Moldova, it will be conducted in youth clinics." Also, please define "youth clinics".

Response: Thank you for the suggestion. Since the recruitment settings differ by country, with North Macedonia primarily recruiting through schools and Moldova through youth clinics, the analyses will also be conducted with country as a variable in the model to consider country level differences. Additionally, the term "youth clinics" has been defined earlier in the manuscript under the study setting: "HYA supports publicly funded youth clinics that deliver preventive and therapeutic services, addressing areas such as sexual and reproductive wellness, psychological well-being, substance misuse, and prevention of violence."

- Page 13, lines 9 (primary outcomes). How were the primary outcomes selected? Why only parent/caregiver reported mental health outcome (CBCL)? Would a self-report assessment of mental health/functioning be warranted?

Response: Thank you for the thoughtful feedback. The decisions of the primary outcomes were informed by findings from the Phase 1 study and the study's theory of change as described in Figure 1. Our age range of youth (10-14) is not completely covered by the threshold for self-report mental health measures (e.g., YSR), which often set the lower threshold of at least 11 years or older. We thus focused on parent/caregiver-reported mental health outcomes (CBCL) as primary outcome.

Nevertheless, adolescent self-reported mental health is included as a secondary outcome to provide a comprehensive evaluation from multiple perspectives. This approach ensures that both caregivers' and adolescents' views are considered.

- How will potential risk (ie disclosure of suicidal thoughts/ideation) be handled during the study? Is there a protocol in place? Will there be clinical support available to the research teams?

Response: Thank you for the feedback. The study includes a detailed protocol to address potential adverse events and risks, such as psychological distress and disclosures of suicidal thoughts or ideation. We have revised the text in the manuscript to enhance clarity: "Staff members will be trained to identify and address adverse events caused by the research or intervention, guided by an adverse event and safety protocol. In case of disclosure of self-harm by a participant, staff will provide immediate risk assessment, support, and referral. All serious adverse events will be reported to the local PI, the study coordinator at the University of Klagenfurt, the responsible ethics boards, and the Data Safety and Monitoring Board (DSMB) for review. The DSMB and ethical boards will independently review the events to decide if any changes to the project are needed. Support is also available for the research team, with guidance provided on how to manage distressing disclosures and access emotional support if required. This is also part of the trainings for project personnel involved in the study."

- Page14, statistical analysis section; please add appropriate citations throughout section (there are none included); including the quantitative, health economics, and qualitative sections.

Response: Thank you for noting this issue. We have included the appropriate citations.

Reviewer #2

- Abstract: Line 19 - Can the term “engagement booster” be better explained or elaborated on?

Response: Thank you. We have revised the abstract to better explain the engagement booster: “…evaluates the effectiveness and costs of three add-on components for the Parenting for Lifelong Health for Parents and Teens program: adolescent mental health tools based on UNICEF’s Helping Adolescents Thrive comics, adolescent peer support based on UNICEF’s I Support My Friends intervention, and an engagement booster designed to enhance attendance and program completion through incentives.”

- Throughout Manuscript: Please ensure the use of numerals <10 (unless applied to a specific phase) are written as words, consistently, throughout the manuscript.

Response: Thank you. We have addressed this issue throughout the manuscript.

- Methods: Page 7 - The role of and training required for supervisors in the program is unclear. Who are these people, what supervision are they agreeing to provide, and what training will they be participating in?

Response: Thank you for the feedback. We agree that clarification on the identity and role of supervisors can strengthen the Methods section. Supervisors are experienced staff

from ALTERNATIVA (North Macedonia) and the Health for Youth Association (HYA, Moldova). These individuals have prior experience in delivering and supporting family-based interventions. Their primary role is to ensure the quality and fidelity of program delivery by providing support, supervision, and feedback to facilitators. Supervisors are required to participate in comprehensive training, which includes facilitation techniques, supervisory methods, and the use of the PLH-Facilitator Assessment Tool (PLH-FAT) to assess and enhance facilitator performance. Some of this information was specified already in the "Intervention" section. Additional text has been added in the revised manuscript to enhance clarity: "The program will be implemented by the current staff and established networks of ALTERNATIVA and HYA. This staff includes data assessors, facilitators, and supervisors. Data assessors are trained research staff responsible for administering participant assessments, ensuring accurate data collection. Facilitators are trained professionals with prior experience in parenting interventions, responsible for delivering the intervention, leading sessions, and guiding participants through skill-building activities. Supervisors are experienced senior staff who provide oversight and support to facilitators, ensure program fidelity, and conduct facilitator performance evaluations. Facilitators, supervisors, and data assessors must be 18 years or older, participate in training, agree to fulfil their respective roles in program delivery, supervision, or assessment, and provide consent."

- Methods: Page 7, Line 45 - Provide an example of home practice tasks to be assigned between sessions.

Response: We agree with providing more details about home task examples. We have revised the text accordingly: "Home practice tasks are assigned between sessions to reinforce learning. For example, after a session on building positive relationships, caregivers and adolescents may asked to spend at least 15 minutes of uninterrupted quality time together

each day, during which the adolescent chooses an activity they enjoy. Caregivers are also encouraged to practice structured praise, offering at least one specific, positive comment to their adolescent each day."

- Methods - Experimental Conditions: A diagram of the stratification of experimental conditions between countries would be worthwhile for clarity and understanding of the methodology.

Thank you for the feedback. We have included a flow chart of the study to enhance clarity.

- Participant Timeline - Page 14: Please update the timeline to reflect the most likely data collection period (i.e., not starting late 2024).

Response: Thank you. We have updated this section: The study implementation timeline varies between North Macedonia and Moldova to accommodate practical considerations unique to each context. Recruitment of potential caregivers and adolescents will begin after cluster randomization, and is scheduled for October 2024 in North Macedonia and December 2024 in Moldova. Recruitment will continue until the target sample size of 320 enrolled families per country is achieved. In North Macedonia, the intervention and related assessments will be conducted in two waves (beginning in October 2024 and January 2025), while one wave will be used in Moldova (beginning in January 2025).

- Ethics and Dissemination - Page 14: Please clarify whether adolescents will be providing consent themselves to engage in the programs, or if this consent will be provided by parents/caregivers.

Response: Thank you for your comment. In both countries, adolescents will provide assent to participate in the program, while their caregivers will provide formal consent. For adolescents aged ≥ 14 in North Macedonia, the adolescents will also be asked to provide consent, as it is mandatory to comply with local legal and ethical requirements. We have revised the text in the manuscript accordingly: "Adolescents must be between 10 and 14 years old at the baseline assessment, and both the caregiver and the teen must agree to participate in the study. In North Macedonia, adolescents aged ≥ 14 are also required to provide consent in order to comply with local legal and ethical requirements."
